# Descriptive study of severe hospitalized cases of laboratory-confirmed influenza during five epidemic seasons (2010–2015)

**DOI:** 10.1186/s13104-018-3349-y

**Published:** 2018-04-14

**Authors:** Núria Torner, Ana Martínez, Luca Basile, MMar Mosquera, Andrés Antón, Cristina Rius, M. Rosa Sala, Sofia Minguell, Elsa Plasencia, Mónica Carol, Pere Godoy, Núria Follia, Irene Barrabeig, M. Angeles Marcos, Tomàs Pumarola, Mireia Jané

**Affiliations:** 1 Public Health Agency of Catalonia, Roc Boronat, 81-95, 08005 Barcelona, Spain; 2 CIBER Epidemiologia y Salud Pública CIBERESP, Madrid, Spain; 3 Departament of Public Health, University of Barcelona, Barcelona, Spain; 4 Microbiology Department, Hospital Clínic de Barcelona, Barcelona, Spain; 5 Microbiology Department, Hospital Universitari Vall d’Hebron de Barcelona, Barcelona, Spain; 6 Public Health Agency of Barcelona, Barcelona, Spain

**Keywords:** Influenza, Surveillance, Epidemiology, Antiviral drugs, Vaccine

## Abstract

**Objective:**

The Plan of Information on Acute Respiratory Infections in Catalonia (PIDIRAC) included the surveillance of severe hospitalized cases of laboratory-confirmed influenza (SHCLCI) in 2009. The objective of this study was to determine the clinical, epidemiological and virological features of SHCLCI recorded in 12 sentinel hospitals during five influenza seasons.

**Results:**

From a sample of SHCLCI recorded during the 5 influenza epidemics seasons from 2010–2011 to 2014–2015, Cases were confirmed by PCR and/or viral isolation in cell cultures from respiratory samples. A total of 1400 SHCLCI were recorded, 33% required ICU admission and 12% died. The median age of cases was 61 years (range 0–101 years); 70.5% were unvaccinated; 80.4% received antiviral treatment (in 79.6 and 24% of cases within 48 h after hospital admission and the onset of symptoms, respectively); influenza virus A [37.9% A (H1N1)pdm09, 29.3% A (H3N2)] was identified in 87.7% of cases. Surveillance of SHCLCI provides an estimate of the severity of seasonal influenza epidemics and the identification and characterization of at-risk groups in order to facilitate preventive measures such as vaccination and early antiviral treatment.

**Electronic supplementary material:**

The online version of this article (10.1186/s13104-018-3349-y) contains supplementary material, which is available to authorized users.

## Introduction

Influenza is an infectious disease affecting mainly upper respiratory tract worldwide. Influenza virus causes between three and five million severe cases and an estimated 250,000–350,000 deaths annually. In the European Union, there are between 40,000 and 220,000 annual deaths attributable to influenza. However, mortality is only the tip of the iceberg in terms of the disease burden, since influenza also causes a decrease in functional status and increased dependency in the elderly [1]. Estimating the burden of disease caused by influenza is difficult because many cases do not require medical care, or no confirmatory laboratory tests are widely performed to all influenza like illness’ cases [2, 3].

## Main text

In Catalonia, influenza surveillance is conducted through the Plan of Information on Acute Respiratory Infections in Catalonia (PIDIRAC) based on the network of sentinel physicians, who provide information on patients with influenza symptoms [4]. Given the situation generated by the 2009 pandemic caused by the new influenza A (H1N1) pdm09 virus, the PIDIRAC sentinel network included surveillance of severe hospitalized cases of laboratory-confirmed influenza (SHCLCI) to assess severity. The PIDIRAC sentinel surveillance network has a primary care sentinel network made up by 60 GPs and pediatricians who inform on a daily basis of all ILI attended and perform sampling of respiratory swabs for confirmation. This information allows to plot weekly ILI incidence and 12 sentinel hospital facilities who notify on a weekly basis all influenza confirmed cases that meet the ECDC definition for severe influenza [5, 6].

This surveillance allows the clinical and epidemiological characteristics and risk factors associated with greater severity to be determined, and the emergence of influenza virus strains with clinical characteristics and behaviours outside the normal range to be detected, in order to correctly prioritize and direct preventive and control measures during the influenza season [7].

The aims of SHCLCI surveillance are to provide an estimate of the severity of seasonal influenza epidemics to identify and characterize the risk groups that may present serious complications as a result of infection by circulating influenza viruses or their association with some underlying diseases and to identify the virological characteristics of viruses associated with these severe cases, such as genetic changes and/or antigenic changes that lead to increased virulence.

The aim of this study was to describe the clinical, epidemiological and virological characteristics of SHCLCI based on data collected in five influenza seasons in Catalonia.

### Methods

Epidemiological surveillance of severe cases of influenza in Catalonia during five epidemic influenza seasons (2010–2015), beginning on week 40 of the season until week 20 of the following year, with the recording by twelve hospitals (covering 95,3% of the population) from the PIDIRAC sentinel network of SHCLCI reported to the epidemiological surveillance units corresponding to each hospital [5, 7].

SHCLCI cases were cases with previous influenza like illness symptoms (sudden onset of symptoms and/or fever; malaise; headache; muscle pain; and/or cough; sore throat; shortness of breath) who presented to a hospital facility and complyed with SHCLCI case definition. SHLCIC was defined as a severe case of laboratory-confirmed influenza due to the influenza virus (A, A (H1N1)pdm09, B, C) that required hospitalization because of pneumonia, septic shock, multiorgan failure or any other severe condition, including ICU admission or who developed clinical signs during hospitalization for other reasons. Influenza diagnosis was confirmed by polymerase chain reaction (PCR) and/or culture of nasopharyngeal swabs. Respiratory tract samples were processed within 24 h of receipt at the laboratory. A 300 μL aliquot was taken for total nucleic acids extraction and eluted in 25 μL of RNase-free elution buffer using the automatic QIAsymphony system (Qiagen, Hilden, Germany) according to the manufacturer’s instructions. Subsequently, two specific one-step multiplex real-time PCR was carried out using the Stratagene Mx3000P QPCR Systems (Agilent Technologies, Santa Clara, CA, USA), were used for typing A/B influenza virus and subtyping influenza A virus [8, 9].

For each reported case, an epidemiological survey was made to collect anonymized demographic variables (age and sex); risk factors; ICU admission; day of onset of symptoms, of hospital admission and discharge; vaccination history; influenza virus type and subtype; and outcome at hospital discharge. Epidemiological survey was conducted by preventive medicine physician from data in medical history registry and public health epidemiologist in charge.

We studied all data on SHCLCI from five influenza seasons in PIDIRAC sentinel network hospitals and made a comparative analysis of viral types and subtypes. The strain identified in > 50% of cases in each season was considered the predominant strain. Duration of hospital stay was divided into two categories < 10 days and 10 days or more. The statistical analysis was made using the Chi square test and Student’s t test with 95% confidence intervals (CI) for continuous variables and the ANOVA test for categorical variables.

### Results

During the 2010–2015 seasons 1400 cases of SHCLCI were recorded, 462 (33%) required ICU admission and 167 (12%) died: 778 (55.6%) were male. The median age was 61 years (range 0–101 years-mean 55.2 (SD 26.7 years). The most-affected age group was the ≥ 65 years age group with 633 cases (45.2%) (Table 1). The median age of the ≥ 65 years age group was 79 years (range 65–101) and the mean age was 78.7 years (SD 7.8 years): 296 (47%) were aged ≥ 80 years. Of deaths, 111 (66.5%) occurred in patients aged ≥ 65 years and 55 (33.3%) in patients aged > 80 years (Table 1).

**Table 1:** Distribution of SHCLCI in Catalonia by age group and influenza virus type-subtype. PIDIRAC 2010–2015

Age group (years)	SHCLCI cases	%	Death/CFR (%)		
0–4	167	11.9	2 (1.2)		
5–14	46	3.3	1 (2.2)		
15–44	172	12.3	10 (5.8)		
45–64	382	27.3	43 (11.3)		
≥ 65	633^a^	45.2	111 (17.5)		
Total	1400		167 (12%)		

All cases type/subtype	Number of cases (%)	Mean age	SD	95% CI lower	95% CI upper
A (H3N2)	410 (29.3)	66.9	23.6	64.6	69.2
A (H1N1)pdm09	531 (37.9)	46.8	25.8	44.6	49.0
B	172 (12.3)	53.7	28.5	49.4	58
Total with type-subtype 1113/1400	1113 (79.5)	55.3	27.1	53.7	56.9

Cases with outcome death type/subtype	Number of cases (%)	Mean age	SD	95% CI lower	95% CI upper
A (H3N2)	54 (38.9)	78.8	11.3	75.7	81.8
A (H1N1)pdm09	62 (44.6)	60.2	17.5	56	64.6
B	23 (16.5)	75.1	13.4	69.4	81
Total with type-subtype 139/167	139 (83.2)	69.8	17.1	67	72.7

p < 0.001

p < 0.001

^a^Distribution of cases aged ≥ 65 years: 296 (46.7%) ≥ 80 years with highest CFR 55/33.3%

The distribution by type of influenza virus was: 87.7% (1228) influenza virus A, 531 (37.9%) of which corresponded to the A (H1N1)pdm09 subtype and 410 (29.3%) to A (H3N2), and 20.5% to influenza A that remained unsubtyped: 172 (12.3%) of cases were influenza B (Additional file 1).

There were significant differences in the mean age of cases according to the virus type, with a higher prevalence of virus A (H3N2) in older patients and virus A (H1N1)pdm09 in younger patients with mean age of cases 66.9 and 46.8 years (p < 0.001) and those with death as outcome 78.8 and 60.2 years, respectively (p < 0.001) (Table 1).

In 1384 (98.9%) of SHCLCI there was a known risk factor. The most prevalent risk factors were cardiovascular disease, chronic obstructive pulmonary disease and diabetes (25.5, 23.4 and 20.5%, respectively). The most prevalent complication was pneumonia in 992 (71.7%) cases, of these 304 (30.6%) presented bacterial superinfection. For cases with known immunization for influenza, 682/967 (70.5%) of cases were not vaccinated for the current season included in the study (missing data on vaccination status: 433 (31%). The age group with highest vaccine coverage was the older than 65 age group (57%) and cases with at least one risk factor had low vaccination coverage (20.5%). Vaccine proved effective in reducing Intensive care unit (ICU) admission [OR = 0.64 (95% CI 0.47–0.88) p = 0.003] (Table 2).

**Table 2:** Distribution of risk factors and complications, clinical characteristics of SHCLCI according to vaccination status, PIDIRAC 2010–2015

Risk factor	(%)	Complications	(%)	
Heart disease	25.5	Pneumonia	71.5	
Asthma	6.5	Bacterial coinfection	30.5	
COPD	22.6	ARDS		
Diabetes	20.5	(Acute respiratory distress syndrome)	37.7	
Immunodeficiency	17.5	Multi organic failure	10.5	
Others (including cancers)	7			
Chronic renal disease	11			
Obesity (BMI ≥ 40)	8			
Chronic liver disease	5.5			
Pregnancy	1.6			
Smoking^a^	37.6			

Clinical characteristic Total cases (%)	Vaccinated^b^ N/cases (%) 285 (29.5%)	Unvaccinated^b^ N/cases (%) 682 (70.5%)	OR (95% CI)	p
Complication pneumonia 998 cases (71.7)	198/284	505/678	0.79 (95% CI 0.58–1.07)	0.07
Bacterial superinfection 309 cases (30.6)	61/188	155/470	0.97 (95% CI 0.68–1.40)	0.58
ARDS 518 cases (37.7)	101/284	205/676	1.20 (95% CI 0.94–1.7)	0.06
Multi organ failure 813 cases (10.1)	31/282	57/671	1.20 (95% CI 0.94–1.7)	0.13
ICU admission 462 cases (33)	71/285	232/682	0.64 (95% CI 0.47–0.88)	0.003

^a^Missing data in 91% of cases

^b^Cases with information. Missing information on immunization status in 433 cases (31%)

Of the 21 pregnant women hospitalized as SHCLCI, all were unvaccinated, 14 (66.7%) required ICU admission, 19 (90.5%) received antiviral treatment and none of them had any underlying disease or risk factor other than pregnancy.

The mean hospital stay was 13.8 days (SD 17.9) with a median of 9 days (range 1–374 days). The mean stay by age group was: 0–4 years 7.44 (SD 8.56 years); 5–14 years 9.24 (SD 7.33 years); 15–44 years 12.37 (SD 14.16 years); 45–64 years 16.41 (SD 17.65 years) and ≥ 65 years: 14.64 (SD 20.64 years). There was a significant difference in the 45–64 years age group, with a mean stay of 16.41 days (SD 17.65 days) (p < 0.001), independently of the viral type and subtype.

A total 1125 cases (80.4%) had information on antiviral treatment, 1113 (99%) received oseltamivir and 12 (1%) zanamivir. 863 of these cases (79.6%) received treatment in the first 48 h after admission. Antiviral treatment administered before 48 h on admission was associated with a shorter length of stay (LOS) (OR 0.25: CI 0.18–0.34, p < 0,001) Nosocomial cases (41) were excluded from the analysis (Table 3).

**Table 3:** Characteristics of length of hospital stay of severe hospitalized laboratory confirmed influenza cases treated and untreated with antivirals, PIDIRAC 2010–2015

NAI treatment^a^	Length of stay < 10 days	Length of stay 10 days and more	Median length of stay 9 days (range 1–374)	
			Crude OR (95% CI)	*p* value
Total	575 (53%)	509 (47%)		
≤ 48 h on admission 863 (79.6%)	529 (92%)	334 (65.6%)	0.17 (0.12–0.24)	< 0.001
> 48 h on admission 221 (20.4%)	46 (8%)	175 (34.4%)		
Total 1084/1400 (77.4%)				

^a^Missing data 275 cases; 41 nosocomial cases excluded

### Discussion

After the 2009 influenza virus A(H1N1)pdm09 pandemic, among the lessons learned was the need to expand surveillance of seasonal influenza to include severe cases in order to determine the characteristics of SHCLCI caused by seasonal influenza viruses circulating during each season.

The results obtained by the PIDIRAC sentinel surveillance system during five post-pandemic seasons underscore the importance of prevention by vaccination in order to avoid serious complications such as ARDS and ICU admission of the most vulnerable persons, while showing the need for increased vaccination coverages in groups such as pregnant women, in whom the proportion of ICU admission is 66.7% while vaccination is zero [7, 10].

In our study no significant differences between influenza A and B virus infections among hospitalized cases was observed, except for younger age for A (H1N1)pdm09 cases; similar results also found by other studies in the United States and Australia [11–13]. Although the number of hospitalizations associated with influenza A virus infections was greater than the number with influenza B virus infections this fact can be explained by greater prevalence of influenza A viruses circulating in the community during the seasons included in the study.

The delay in the administration of antiviral drugs at symptom onset in people with an identified risk of complications, such as the elderly or people with medical conditions that worsen the prognosis of influenza or make a longer hospital stay likely, also demonstrates the need to confirm influenza in primary healthcare and administer treatment within 48–72 h for it to be effective.

Influenza remains an important global public health problem in spite of scientific evidence which support immunization to protect those at high risk for complications, such as the elderly [1]. Predominant influenza type/subtype circulating each season, influenza vaccination policies and coverage, influenza vaccine strain match/mismatch and vaccine effectiveness significantly influence the % of hospitalised influenza cases and CFRs in all age groups, including older age groups. However, the high percentage of hospitalizations (45.2%) and mortality (17.5%) in the ≥ 65 years age group, especially in people aged > 80 years, where mortality is higher (33.3%), reflect the consequences of increased life expectancy. Early administration of antiviral treatment has proven to diminish length of stay. Healthcare providers should start antiviral treatment as soon as possible, before 48 h from onset of symptoms is the recommendation, [11] unfortunately this is not feasible. Yet if treated as soon as patient is admitted to the hospital facility and influenza is confirmed, shorter length of stay and prompt recovery can be attained [12, 13].

This makes it necessary to deepen our knowledge of the effect of aging and its interaction with the most prevalent chronic diseases in the elderly and the immune response in order to implement preventive measures to provide better protection of this population group [11].

It is necessary to improve some surveillance aspects, especially with regard to data collection, in order to avoid a loss of information that makes some variables impossible to assess, such as risk factors such as smoking, which was not recorded in 91% of cases as well as lack of information on the vaccination status, which was more than 30% [14, 15].

## Limitations

A limitation to this study is that only SHCLCI cases were recorded during the study period. This unables global hospitalization burden estimates caused by seasonal influenza nor the estimation of seasonal differences in vaccine effectiveness to prevent severity and death. The system identifies the epidemiological and virological characteristics of severe forms of influenza that show changes in their virulence, but comparison between severe and non-severe cases is not feasible. The proportion of SHCLCI cases admitted to ICU and CFRs are potentially higher than other surveillance systems that monitor all hospitalised cases of confirmed influenza. This is particularly evident with regard to pregnant women because of the small number of cases.

Yet, in all SHCLCI surveillance provides an estimate of the severity of seasonal influenza epidemics, and provides ad hoc information to identify and characterize the groups at risk of complications and take appropriate preventive measures.

## Additional file

**Additional file 1.** SHLCI 2010_2015. Information on cases assessed in the study of severe influenza laboratory confirmed hospitalized cases. Catalonia (Spain) from 2010–2011 to 2014–2015.

## Abbreviations

ARDS: acute respiratory distress
ICU: intensive care unit
CFR: case fatality rate
CI: confidence interval
ECDC: European Center for Disease Control
ILI: influenza like illness
LOS: length of stay
OR: odds ratio
PCR: polymerase chain reaction
PIDIRAC: Plan of Information on Acute Respiratory Infections in Catalonia
SD: standard deviation
SHCLCI: severe hospitalized cases of laboratory-confirmed influenza
